# Neurosensory Disturbances Following Inferior Alveolar Nerve Relocation and Implant Placement: A Systematic Review and Meta-Analysis

**DOI:** 10.3390/jcm14165741

**Published:** 2025-08-14

**Authors:** Raffaele Vinci, Saverio Cosola, Korath Varkey M, Sowndarya Gunasekaran, Jaibin George, Ugo Covani

**Affiliations:** 1 Post-Graduate School of Oral Surgery, Dental School, Vita-Salute and IRCCS San Raffaele University, 20132 Milan, Italy; vinci.raffaele@hsr.it; 2 Department of Stomatology, Tuscan Stomatologic Institute, Foundation for Dental Clinic, Research and Continuing Education, 55041 Camaiore, Italycovani@covani.it (U.C.); 3 Paulose Memorial Dental Clinic & Maxillofacial Surgery Centre, Fairlands, Salem 636016, India; 4 Vinayaka Mission’s Sankarachariyar Dental College, Vinayaka Mission’s Research Foundation, Salem 636010, India

**Keywords:** dental implants, inferior alveolar nerve, jaw, edentulous, partially, mandibular atrophy, neurosensory disorders, nerve transfer, sensory receptor recovery, surgical flaps

## Abstract

**Background:** Rehabilitation of atrophic posterior mandibles using dental implants is often complicated by anatomical limitations, particularly the proximity of the inferior alveolar nerve (IAN). Techniques such as IAN lateralization and transposition enable implant placement but are associated with neurosensory disturbances (NSDs). This systematic review and meta-analysis aimed to assess the incidence, duration, and predictors of NSDs following IAN repositioning for implant placement and to evaluate the effectiveness of adjunctive methods like piezo-surgery and platelet-rich fibrin (PRF) in minimizing complications. **Methods:** Following PRISMA 2020 guidelines, a comprehensive search of electronic databases and gray literature identified 20 studies, including randomized controlled trials, prospective cohorts, and retrospective analyses published between 2009 and 2024. Outcomes analyzed included incidence of NSDs, recovery rates, implant stability quotient (ISQ), marginal bone loss, and implant success rates. Meta-analysis was performed using RevMan 5.3 software, with heterogeneity and publication bias assessed using standard Cochrane tools. **Results:** Transient NSDs occurred in 15–40% of cases, with higher rates observed in transposition techniques. Most patients experienced recovery within 6 months. Piezoelectric surgery significantly reduced the incidence and duration of NSDs compared to rotary instruments. Meta-analysis revealed no statistically significant differences between lateralization and transposition techniques in ISQ, marginal bone loss, success rate, or NSDs at 3 months (*p* > 0.05). PRF was associated with accelerated nerve recovery. IAN repositioning is effective for implant placement in atrophic mandibles with a risk of transient NSDs. **Conclusions:** Lateralization combined with piezo-surgery and PRF shows favorable outcomes in minimizing nerve injury and optimizing implant success. The PROSPERO registration code is as follows: CRD420251086835.

## 1. Introduction

Rehabilitating edentulous posterior mandibles with Osseo-integrated implants is a complex process, primarily due to the anatomical constraints posed by the inferior alveolar nerve (IAN). Severe mandibular atrophy often limits the available bone height above the nerve, making it challenging to place standard implants without risking neural damage. This has led to the development of alternative strategies, including the use of short implants, vertical bone augmentation, and nerve repositioning techniques such as inferior alveolar nerve lateralization (IANL) and transposition [1,2].

Despite these alternatives, nerve relocation remains relevant in cases of severe mandibular atrophy (less than 4 mm vertical bone) due to the impossibility of placing a short or narrow implant to avoid complex bone regeneration, in cases of previous graft failures, or when augmentation is contraindicated. In such patients, nerve lateralization or transposition allows placement of adequately sized implants while optimizing prosthetic outcomes.

IANL, which involves mobilizing the nerve laterally to create sufficient space for implant placement, has gained prominence as a viable solution for managing atrophic mandibles. This technique allows for the insertion of longer implants, which can help enhance their primary stability and optimize the biomechanical properties of the prosthetic restoration [3,4]. However, despite these advantages, IANL is associated with significant risks of neurosensory disturbances (NSDs), including paresthesia, hypoesthesia, and anesthesia, resulting from nerve manipulation [2,4].

While the long-term success and survival rates of implants placed with IANL are generally high, the potential for mandibular fractures and residual sensory deficits necessitates a careful evaluation of patient selection and surgical expertise [4,5]. Emerging techniques and modifications, such as repositioning the buccal cortical bone or incorporating guided bone regeneration, help enhance favorable outcomes of IANL procedures [1,6].

This systematic review and meta-analysis have the objective of evaluating the incidence, duration, and predictors of NSDs following IANL and implant placement in the posterior mandible. It seeks to provide evidence-based recommendations for the question regarding optimizing clinical outcomes and mitigating complications associated with this procedure.

In this study, we distinguish between two nerve relocation procedures: lateralization (displacement of the IAN without cutting the mental nerve) and transposition (repositioning of the IAN involving the transection and relocation of the mental foramen). Given their different anatomical and clinical implications, we analyzed these procedures separately throughout the review.

Our hypotheses were as follows: (1) that transposition is associated with a higher incidence and delayed recovery of neurosensory disturbances compared to lateralization, and (2) that adjunctive measures such as piezo-surgery and/or PRF (platelet-rich fibrin) may reduce the incidence and duration of nerve disturbances following IAN relocation.

## 2. Materials and Methods

### 2.1. Study Design

The protocol of this systematic review has been registered on PROSPERO with the following code: CRD420251086835.

This study is a systematic review and meta-analysis evaluating neurosensory disturbances (NSDs) in the posterior mandibular region following inferior alveolar nerve repositioning and implant placement. The review adheres to PRISMA 2020 Checklist (Preferred Reporting Items for Systematic Reviews and Meta-Analyses) guidelines to ensure transparency and reproducibility of the methods [7].

The focus question was formulated using the PICOS framework:

1. Population:

Adults who underwent implant placement in the posterior mandibular region after lateralization or transposition of the inferior alveolar nerve (IAN) and presented with neurosensory disturbances.

2. Intervention:

Implant placement in the posterior mandible involving inferior alveolar nerve repositioning using piezosurgery.

3. Comparison:

Implant placement in the posterior mandible involving IAN repositioning using conventional surgical methods.

4. Outcomes:
○ Primary Outcome: Incidence of neurosensory disturbances (e.g., paresthesia, hypoesthesia, or dysesthesia) following IAN repositioning.
○ Secondary Outcome: Rate and extent of spontaneous recovery from neurosensory disturbances following IAN repositioning.
5. Study Design:

Included prospective cohort studies, randomized controlled trials (RCTs), and observational studies.

### 2.2. Eligibility Criteria

#### 2.2.1. Inclusion Criteria

1. Prospective cohort studies, observational studies, and RCTs reporting altered sensation following implant placement.
2. Studies providing data on the onset and duration of neurosensory disturbances postoperatively.
3. Studies published between 2009 and 2024 to capture advancements in techniques.
4. Articles written in English.

#### 2.2.2. Exclusion Criteria

1. Case reports, cross-sectional studies, and literature reviews.
2. In vitro studies, finite element analyses, and animal studies.
3. Studies not meeting the focus on neurosensory disturbance or inferior alveolar nerve repositioning.

### 2.3. Search Strategy

A comprehensive electronic search was performed in the following databases:

1. PubMed;
2. Cochrane Library;
3. Science Direct;
4. Google Scholar;
5. Ovid;
6. Embase;
7. Open Gray (for gray literature).

### 2.4. Keywords and Search Terms

The search strategy employed a combination of MeSH terms and free-text keywords:

- Altered sensation;
- Dental implant;
- Dysesthesia;
- Hyperalgesia;
- Implant placement
- Inferior alveolar nerve;
- Mandibular nerve;
- Nerve injury;
- Nerve lateralization;
- Nerve repositioning;
- Neurosensory disturbance;
- Paresthesia;
- Sensory disturbance;
- Transposition.

### 2.5. Selection Process

The search was carried out by two independent reviewers (K.V.M. and S.C.). Any disagreement between the reviewers, such as whether a study met the inclusion criteria or disagreements on data collection decisions or quality assessment outcomes, was resolved by consensus. The same reviewers read the full texts of the studies selected, including those in which the abstract supplied insufficient information for reaching a decision. Any disagreement between the two reviewers was resolved by discussion with a third reviewer (J.B).

### 2.6. Data Collection Process

A standardized data extraction form was used to record study characteristics:

1. Author and year;
2. Study design;
3. Sample size;
4. Statistical analysis methods;
5. Radiographic method;
6. Method of nerve repositioning/surgical technique;
7. Number of patients with altered sensation;
8. Method of evaluation of altered sensation;
9. Nature of altered sensation;
10. Recovery rate and intervals;
11. Implant survival rate.

Data extraction was performed independently by one author (K.V.M.) and cross-verified by a second author (S.C.) to ensure accuracy and completeness.

### 2.7. Data Items

This is the list of outcomes for which each study was analyzed.

The primary outcomes were as follows: implant stability quotient (ISQ); marginal bone loss; implant success rate; and neurosensory disturbances at 3 months following IAN repositioning (paresthesia, hypoesthesia, and dysesthesia).

The secondary outcomes were as follows: spontaneous recovery rates and durations of neurosensory disturbances postoperatively.

Study risk of bias assessment

- Randomized Studies: Assessed using the Cochrane RoB-2 tool [8].
- Non-Randomized Studies: Assessed with the ROBINS-I tool [9].
- Retrospective Studies: Assessed with the Newcastle–Ottawa Scale (NOS) [10].

### 2.8. Effect Measures

The standardized mean difference (SMD) and odds ratio (OR) with 95% CI were calculated for dichotomous outcomes. A fixed-effects model (Mantel–Haenszel method) was used if there was no heterogeneity (*p* > 0.05 or *I-squared* ≤ 24%); otherwise, a random effects model (Der Simonian–Laird method) was used. All statistical analyses were performed using RevMan 5.3 (Cochrane Collaboration, Software Update, Oxford, UK) [11].

### 2.9. Synthesis Methods

The significance of any discrepancies in the estimates of the treatment effects of the different trials was assessed by means of Cochran’s test for heterogeneity and I2 statistics, which describe the percentage of the total variation across studies that is due to heterogeneity rather than chance. Heterogeneity was considered statistically significant if *p* < 0.1. A rough guide to the interpretation of I^2^ given in the Cochrane handbook is as follows: (1) from 0 to 40%, the heterogeneity might not be important; (2) from 30% to 60%, it may represent moderate heterogeneity; (3) from 50% to 90%, it may represent substantial heterogeneity; (4) from 75% to 100%, there is considerable heterogeneity [12].

### 2.10. Reporting Bias Assessment

To test for the presence of publication bias, the relative symmetry of the individual study estimates was assessed around the overall estimates using Begg’s funnel plot. A funnel plot (plot of the effect size versus standard error) was drawn. Asymmetry of the funnel plot may indicate publication bias and other biases related to sample size, although asymmetry may also represent a true relationship between trial size and effect size [12,13].

### 2.11. Certainty Assessment

The significance level was kept at *p* < 0.05.

### 2.12. Ethical Considerations

As this meta-analysis is based on previously published studies, no ethical approval was required. However, ethical considerations from each included study were recorded.

## 3. Results

### 3.1. Study Selection

**Figure 1:**
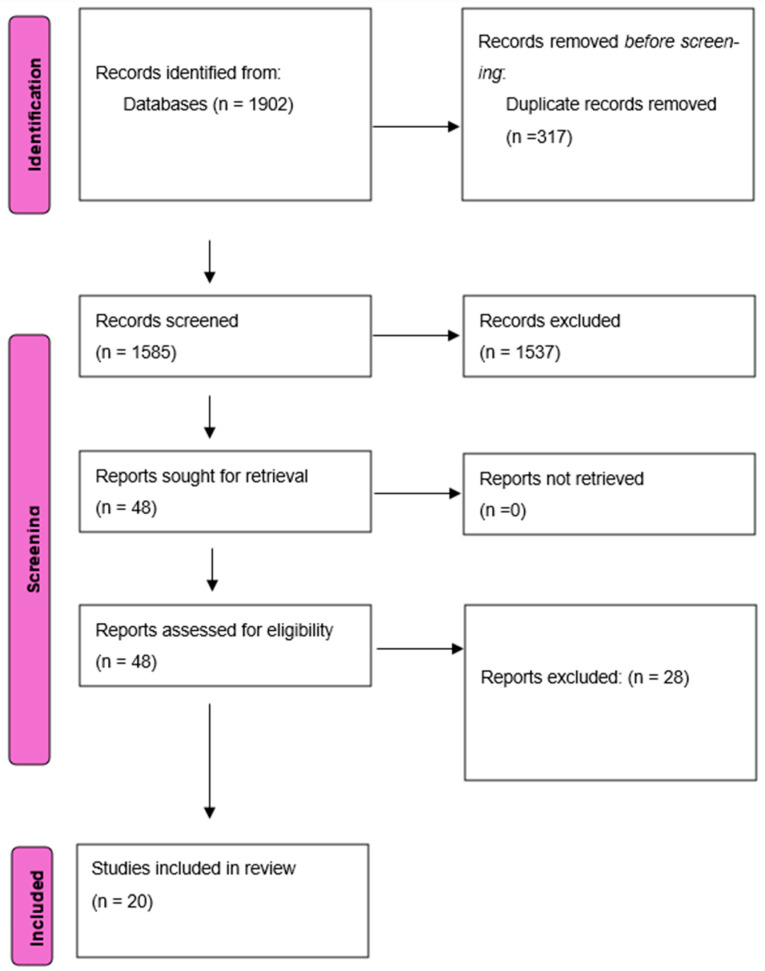
PRISMA flow diagram data.

A comprehensive search of electronic databases and manual searches retrieved a total of 1902 studies. Before screening, 317 duplicates were removed. After screening titles and abstracts, 1537 studies were excluded due to irrelevant titles. Following full-text assessment for eligibility, 20 studies [1,3,6,14,15,16,17,18,19,20,21,22,23,24,25,26,27] met the inclusion criteria and were included in this systematic review. Of these, 13 were prospective studies, and the remaining 7 were retrospective observational studies. Of the 13 prospective studies, 5 were randomized, and the remaining 8 were non-randomized. A PRISMA flow diagram (Figure 1) provides a visual summary of the study selection process [7].

### 3.2. Study Characteristics

The included studies comprised a total of five randomized controlled trials (RCTs), eight prospective studies, and seven retrospective observational studies. Sample sizes varied considerably, ranging from as few as 7 to as many as 123 patients per study. While demographic details such as age and gender were occasionally reported, there was notable inconsistency across studies in providing this information.

The duration of follow-up also varied widely, from a minimum of 4 weeks to a maximum of 5 years, depending on the study design and objectives. In terms of imaging modalities, cone-beam computed tomography (CBCT) was the most commonly employed radiographic tool, followed by panoramic radiographs and conventional CT scans.

Various techniques were used for inferior alveolar nerve (IAN) repositioning. Lateralization was the most frequently adopted method, while others utilized transposition or modified approaches incorporating adjunctive measures such as platelet-rich fibrin (PRF) conduits or piezoelectric surgical devices. Additionally, several studies included the use of bone grafts, sticky bone mixtures, or collagen membranes in conjunction with nerve repositioning procedures.

The evaluation of neurosensory disturbances (NSDs) relied on both subjective and objective methods. Subjective assessments primarily involved patient-reported outcomes, using tools such as questionnaires and the Visual Analog Scale (VAS). Objective evaluation techniques included two-point discrimination (TPD), light touch (LT), brush direction detection (BDD), transcutaneous electrical stimulation potential (TSEP), Von Frey hair testing, and the Modified Nerve Block Recovery (MNBR) scale. These diverse assessment strategies provided comprehensive insights into the extent and resolution of sensory changes following nerve manipulation.

Table 1 provides a detailed summary of study characteristics.

### 3.3. Risk of Bias in Studies and Reporting Biases

The quality of studies included in this systematic review was evaluated using different tools based on the study design. Each type of study was assessed with the most appropriate risk of bias (RoB) tool to ensure comprehensive evaluation.

#### 3.3.1. Randomized Controlled Trials (RCTs)

Assessment Tool: RoB 2 (Risk of Bias 2) reported in Table 2.

**Figure 2:**
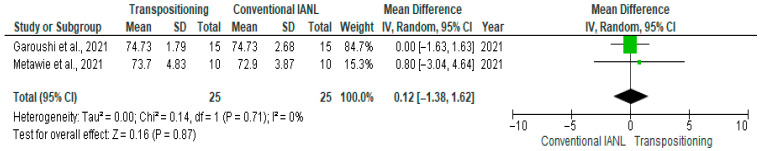
Implant stability quotient between transpositioning and conventional inferior alveolar nerve lateralization of Garoushi et al., 2021 [17] and Metawie et al., 2021 [16].

Findings: The risk of bias (RoB) assessment for the included studies showed consistently low risk across all domains. Abdo et al. [14] demonstrated low risk of bias in all areas, with predefined outcomes reported transparently, strict adherence to the study protocol, and no evidence of selective reporting. Similarly, Campos et al. [15] reported results comprehensively, aligning with study objectives and pre-specified plans, with no deviations observed. Metawie et al. [16] ensured that all pre-specified outcomes were reported without omissions or discrepancies between planned and reported results, minimizing reporting bias. Garoushi et al. [17] also maintained transparency by presenting detailed statistical analyses and outcomes consistent with the original protocol, with no selective reporting concerns (Figure 2). Lastly, Chehata et al. [2] reported all predefined outcomes clearly and consistently as per the study design, with no evidence of incomplete or biased reporting. As a result, all studies were graded as having low risk of bias across all domains, leading to an overall low risk of bias assessment.

#### 3.3.2. Retrospective Studies

Assessment Tool: Newcastle–Ottawa Scale (NOS) reported in Table 3.

Findings: The risk assessment for the retrospective studies, which is based on the Newcastle–Ottawa Scale (NOS), evaluates three key domains: selection, comparability, and outcome. The total score ranges from 0 to 9, with higher scores indicating lower risk of bias.

Lorean et al. [4] and Sethi et al. [18] both received the highest total score of 9/9, categorizing them as low risk. These studies scored well across all domains, with Lorean et al. [4] benefiting from a well-defined and representative cohort, robust adjustments for confounders, and comprehensive follow-up. Sethi et al. also had a large, representative cohort, thorough reporting of outcomes, and minimal loss to follow-up, further enhancing the study’s quality [18].

Castellano et al. [26], Gasparini et al. [3], and Nishimaki et al. [27] all scored 8/9, which is also classified as low risk, but with slight limitations. Castellano et al. [26] had a strong cohort selection but lacked adequate adjustment for confounders, which slightly lowered their comparability score. Gasparini et al. [3] demonstrated strong cohort selection and confounder adjustment, though some issues with unclear follow-up reporting reduced its outcome score. Similarly, Nishimaki et al. [27] had a well-defined cohort and proper confounder adjustments, but incomplete follow-up reporting slightly impacted their outcome score.

George Deryabin et al. [6] and Khojasteh et al. [5] both received scores of 6/9, placing them in the moderate-risk category. George Deryabin et al. [6] had a smaller, less representative cohort and minimal confounder adjustment, which impacted both the selection and comparability domains. While follow-up was adequate, these limitations led to a moderate-risk classification. Khojasteh et al. [5] faced similar issues, with a small sample size and limited confounder adjustments, though follow-up was satisfactory, contributing to its moderate risk score.

Authors should discuss the results and how they can be interpreted from the perspective of previous studies and of the working hypotheses. The findings and their implications should be discussed in the broadest context possible. Future research directions may also be highlighted.

#### 3.3.3. Non-Randomized Studies

Assessment Tool: The ROBINS-I (risk of bias in non-randomized studies) results are reported in Table 4.

Findings: Bayram et al. [19] are classified as having a low risk of bias across all domains. Their study exhibited strong control of confounding variables, clear cohort selection criteria, adherence to intervention protocols, and reliable outcome measurements. Missing data were appropriately handled, and there was no evidence of selective reporting, making it a highly robust study.

De Vicente et al. [1] had a moderate risk of bias, mainly due to limited control over confounding factors and some protocol deviations. However, the study had minimal selection bias through consecutive sampling, and the intervention classification and reporting were clear and transparent. The moderate confounding bias and protocol deviations contributed to an overall moderate risk.

Erhan Dursun et al. [20] presented a moderate risk of bias, with notable issues in several domains. The study had moderate confounding and selection bias, with unclear adjustments for confounders and unclear selection processes. Additionally, moderate subjectivity in outcome measurements and deviations from the intervention protocol further increased the risk. Missing data and reporting bias also contributed to the moderate overall risk.

Fernandez Diaz et al. [21] stand out with low risk of bias across all domains. The study demonstrated thorough control of confounders, clear selection criteria, protocol adherence, and reliable outcome measurements. There were no significant issues with missing data or selective reporting, positioning this study as one with minimal risk of bias.

Hashemi et al. [22] displayed a moderate risk of bias due to insufficient adjustments for confounders and unclear selection criteria. There were also some deviations from the planned protocol and moderate subjectivity in the measurement of outcomes. While missing data were managed reasonably well, some reporting bias was noted, leading to an overall moderate risk classification.

In Rathod et al. [24] and Saad Al-Almaie et al. [25], the overall risk of bias was moderate as well due to similar issues with confounding, selection bias, and protocol deviations. Both studies had subjective outcome measurements and moderate concerns regarding missing data. Reporting bias was also present, though both studies were generally transparent in their reporting.

The full texts of 28 excluded studies are listed in Table 5 [28,29,30,31,32,33,34,35,36,37,38,39,40,41,42,43,44,45,46,47,48,49,50,51,52,53,54,55].

### 3.4. Results of Syntheses

The studies employed a variety of statistical methods to analyze neurosensory disturbances and recovery outcomes:

- Chi-square tests and *t*-tests were the most common, reported in 12 studies [2,3,5,6,14,15,19,21,22,23,24,26] for group comparisons.
- ANOVA was used in four studies [15,17,18,26] to assess variance across multiple groups.

The Mann–Whitney U test was used to compare groups, Spearman’s correlation coefficient was applied to assess relationships, and Fisher’s exact test was used to evaluate significance [1].

Advanced statistical models, including generalized estimating equations (GEEs) and Kaplan–Meier survival analysis, were employed in three studies [17,20,25].

### 3.5. Incidence of Neurosensory Disturbances in Lateralization and Transposition Techniques

- Lateralization of the inferior alveolar nerve (IAN) was evaluated in 12 studies [1,2,5,6,17,18,22,24,25,26,27]. The overall incidence of transient neurosensory disturbances ranged between 15% and 30%, as reported in seven studies [1,2,18,22,24,25,26].
- Transposition techniques were assessed in eight studies [3,5,6,15,20,21,24,26]. Transposition exhibited a higher incidence of neurosensory disturbances, ranging from 25% to 40%, as noted in six studies [3,15,20,21,24,26].

### 3.6. Recovery Timelines Were as Follows:

- Within 4 months: Recovery rates of up to 90% were reported in five studies [1,2,18,22,25].
- Within 6 months: Recovery rates of 75% were observed in four studies [3,15,21,26], reflecting the increased degree of nerve manipulation associated with transposition techniques [3,15,21,26].
- For other studies, the recovery time varied from 6 to 12 months [4,5,6,17,19,20,21,23,24,26,27].

### 3.7. Piezo Surgery vs. Rotary Instruments

Piezosurgery was utilized in 14 studies [1,2,3,4,5,6,14,15,21,22,23,24,25,26] and demonstrated significant advantages, including precise nerve manipulation and reduced thermal damage.

Rotary instruments were employed in six studies [2,3,6,20,21,24], and they were noted for their broader availability and shorter operation times but were associated with higher neurosensory disturbance rates.

### 3.8. Key Findings

The transient neurosensory disturbance rates are as follows:

○ Piezo surgery: 15% to 30% [2,3,5,6,14,15,19,21,22,23,24,25,26];
○ Rotary instruments: 25% to 40% [3,21];
  - Recovery timelines;
○ Piezo surgery: Most patients recovered within 8 weeks to 3 months;
○ Rotary instruments: Recovery extended to 6 to 12 months [14,23].

### 3.9. Implant Success Rate

The overall implant success rate across all studies remained consistently high:

Piezosurgery studies reported success rates between 92% and 100%.
Rotary instrument studies reported slightly lower rates, ranging from 90% to 95%.
Factors influencing implant success included surgical technique, the use of bone grafts, and follow-up duration [5,14,15].

### 3.10. Influence of Bone Grafts and Interface Materials

Seven studies examined the role of bone grafts and interface materials in nerve repositioning:

- Bone Grafts: Autogenous bone grafts enhanced structural stability and supported osseointegration [5,15].
- Interface Materials: PRF and collagen membranes were used to protect nerves from direct implant contact and promote regeneration through the release of growth factors such as the following:
○ Platelet-derived growth factor;
○ Transforming growth factor-beta (TGF-β).

### 3.11. Certainty of Evidence

- PRF significantly accelerated nerve recovery, with 85% of patients regaining sensation within 4 months [5]
- Bone grafting had no significant effect on neurosensory outcomes, as recovery occurred within 6 months for both grafted and non-grafted cases [15]

### 3.12. Meta-Analysis

The effectiveness was assessed and evaluated in terms of implant stability quotient (ISQ), marginal bone loss, success rate and neurosensory disturbances at 3 months, as shown in the figure below.

#### 3.12.1. Implant Stability Quotient (ISQ)

**Figure 3:**
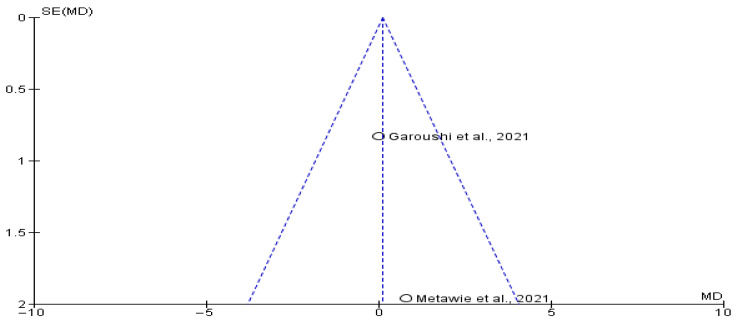
Funnel plot showing absence of possible publication bias of Garoushi et al., 2021 [17] and Metawie et al., 2021 [16].

**Figure 4:**
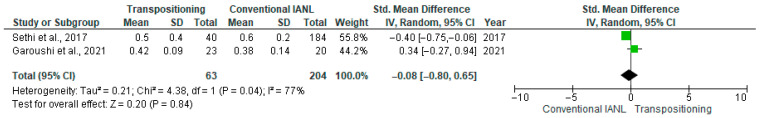
Marginal bone loss between transpositioning and conventional inferior alveolar nerve lateralization of Garoushi et al., 2021 [17] and Sethi et al., 2017 [18].

Two studies [16,17] involving a total of 50 patients (25 undergoing transposition and 25 undergoing inferior alveolar nerve lateralization) were included in the analysis of the implant stability quotient. The standardized mean difference (SMD) was 0.12 (95% CI: −1.38 to 1.62; *p* > 0.05), indicating no statistically significant difference between the groups. The slight numerical advantage in the transposition group was minimal and unlikely to be clinically meaningful. This suggests that implant stability is comparable between both nerve relocation techniques and may depend more on surgical precision than on the choice of nerve repositioning method. The funnel plot analysis (Figure 3 and Figure 4) showed no evidence of publication bias.

#### 3.12.2. Marginal Bone Loss

**Figure 5:**
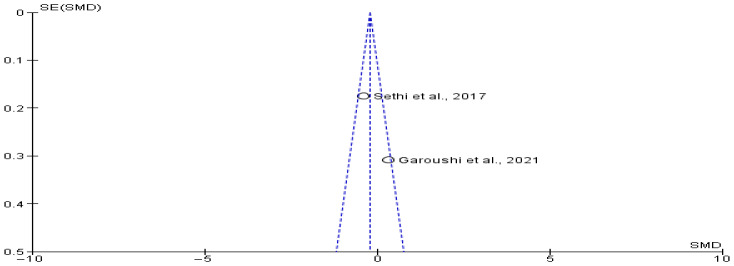
Funnel plot showing absence of possible publication bias of Garoushi et al., 2021 [17] and Sethi et al., 2017 [18].

Marginal bone loss outcomes were extracted from two studies [17,18], encompassing 267 patients (63 in the transposition group and 204 in the lateralization group). The meta-analysis revealed an SMD of −0.08 (95% CI: −0.80 to 0.65; *p* > 0.05), indicating that marginal bone loss was slightly lower in the transposition group. However, this difference was not statistically significant. The observed variance might reflect differing bone remodelling responses or surgical exposure rather than the nerve repositioning technique itself. The funnel plot analysis (Figure 5) did not suggest any publication bias.

#### 3.12.3. Implant Success Rate

**Figure 6:**
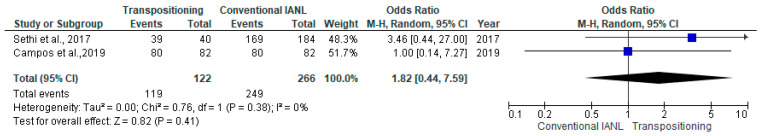
Implant success rate between transpositioning and conventional inferior alveolar nerve lateralization of Campos et al., 2019 [15] and Sethi et al., 2017 [18].

**Figure 7:**
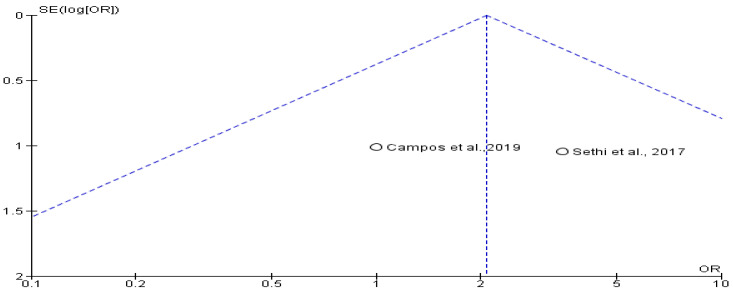
Funnel plot showing absence of possible publication bias of Campos et al., 2019 [15] and Sethi et al., 2017 [18].

The implant success rate was evaluated in 388 patients across two studies [15,18], with 122 undergoing transposition and 266 undergoing lateralization. The pooled odds ratio (OR) was 1.82 (95% CI: 0.44 to 7.59; *p* > 0.05), indicating that the transposition group had higher odds of implant success, though the difference was not statistically significant. The wide confidence interval and overlapping values reflect variability between study populations and procedures. No evidence of publication bias was found (Figure 6 and Figure 7).

#### 3.12.4. Neurosensory Disturbances at 3 Months

**Figure 8:**
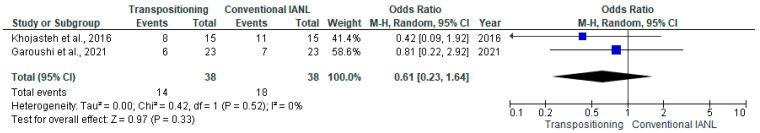
Neurosensory disturbances at 3 months between transpositioning and conventional inferior alveolar nerve lateralization of Garoushi et al., 2021 [17] and Khojasteh et al., 2016 [5].

**Figure 9:**
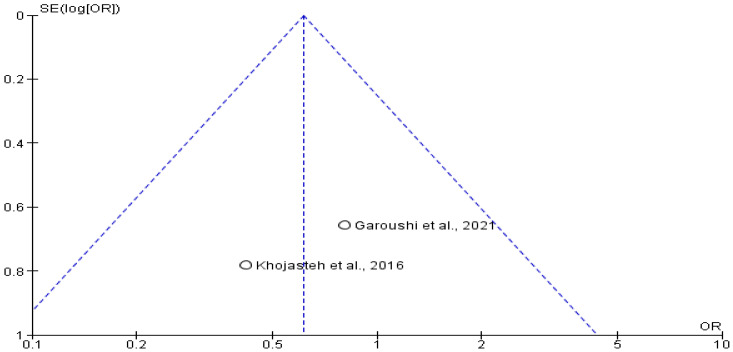
Funnel plot showing absence of possible publication bias of Garoushi et al., 2021 [17] and Khojasteh et al., 2016 [5].

Data on short-term neurosensory outcomes were available from two studies [15,18], including 32 patients (14 in the transposition group and 18 in the lateralization group). The odds ratio for neurosensory disturbances at 3 months was 0.61 (95% CI: 0.23 to 1.64; *p* > 0.05), favoring the transposition group, although the result was not statistically significant. This trend may reflect individual patient variation or small sample sizes. The corresponding funnel plot (Figure 8 and Figure 9) did not reveal any major publication bias.

## 4. Discussion

### 4.1. Atrophic Mandibular Rehabilitation

Rehabilitation of the atrophic mandible, particularly the posterior region, is a complex clinical challenge due to reduced vertical bone height and the proximity of the inferior alveolar nerve (IAN) [28]. These anatomical limitations increase the complexity of surgical interventions and the risk of complications. Despite these challenges, advancements in surgical techniques, technology, and implantology have improved treatment outcomes. This discussion outlines the key challenges faced and compares the most commonly used techniques for managing atrophic mandibles, including nerve lateralization, transposition, and vertical bone augmentation.

### 4.2. Challenges in Atrophic Mandibular Rehabilitation

The primary challenge in rehabilitating the atrophic mandible lies in the insufficient bone volume and vertical height, which complicates the placement of short dental implants. The close proximity of the inferior alveolar nerve further complicates the situation, as the nerve must be protected during surgical procedures to avoid irreversible neurosensory disturbances, such as paresthesia, anesthesia, or dysesthesia, which can significantly impact the quality of life of patients and complicate postoperative recovery [26,27,28,29,30,56]. Additionally, vertical bone loss in the posterior mandible is often accompanied by decreased bone density, reducing the possibility of stable implant placement without prior bone augmentation. This presents additional challenges in terms of surgical planning, patient selection, and the need for advanced imaging technologies like cone-beam computed tomography (CBCT) to accurately assess bone volume and nerve location [29].

### 4.3. Implant Stability Quotient (ISQ)

The pooled analysis from two studies with 50 samples revealed that the standardized mean difference (SMD) was 0.12 (−1.38 to 1.62), indicating no statistically significant difference between transpositioning and inferior alveolar nerve lateralization methods in terms of ISQ (*p* > 0.05). This suggests that both techniques are comparable in achieving implant stability, a critical factor for the success of dental implants. The absence of publication bias further strengthens the reliability of these findings [16,17].

### 4.4. Marginal Bone Loss

Marginal bone loss is a crucial parameter in evaluating the long-term success of dental implants. The meta-analysis of two studies with 267 samples showed an SMD of −0.08 (−0.80 to 0.65), indicating that transpositioning led to slightly lesser bone loss compared to the inferior alveolar nerve lateralization approach.

However, this difference was not statistically significant (*p* > 0.05). These results suggest that both surgical techniques have similar impacts on preserving marginal bone, and neither demonstrated a distinct advantage over the other [17,18].

### 4.5. Success Rate

The success rate of the two approaches was analyzed based on data from 388 samples. The odds ratio (OR) was 1.82 (0.44 to 7.59), suggesting that the transpositioning group had 1.82 times higher odds of success compared to the inferior alveolar nerve lateralization approach. However, this difference did not reach statistical significance (*p* > 0.05). These findings imply that while there may be a trend favoring transpositioning, further research with larger sample sizes is necessary to confirm this observation [15,18].

### 4.6. Neurosensory Disturbances at 3 Months

Neurosensory disturbances are a common concern following procedures involving the inferior alveolar nerve. The analysis of 32 samples showed an OR of 0.61 (0.23 to 1.64), indicating a trend towards fewer neurosensory disturbances in the transpositioning group. However, this difference was not statistically significant (*p* > 0.05). These findings align with the overall trend of no significant differences between the two approaches in terms of safety outcomes [5,17].

### 4.7. Heterogeneity and Publication Bias

The assessment of heterogeneity using Cochran’s test and *I^2^* statistics revealed no significant heterogeneity in the included studies. Additionally, Begg’s funnel plots for all outcomes showed no evidence of publication bias, enhancing the credibility of the meta-analysis.

### 4.8. Techniques for Mandibular Nerve Management

Several techniques have been developed to address these challenges, with lateralization and transposition of the IAN being the most commonly used approaches. Both techniques aim to preserve the nerve’s function while providing sufficient space for implant placement.

#### 4.8.1. Nerve Lateralization

Lateralization of the IAN involves repositioning the nerve laterally to increase the available space for implant placement. Studies have shown that lateralization has a high success rate, with implant survival rates ranging from 93.8% to 100%. Additionally, 90% or more of patients experience significant sensory recovery within 6 months, often recovering as early as 3 months [14,15]. This technique offers a promising solution for posterior mandible rehabilitation as it is less invasive and results in faster recovery compared to other methods. The success of lateralization largely depends on surgical technique and the use of advanced tools like piezosurgery, which offers greater precision with minimal damage to surrounding tissues [3]. However, complications such as mandibular fractures, although rare, have been reported in some studies.

#### 4.8.2. Nerve Transposition

Transposition involves repositioning the IAN to a more favorable position, typically further anteriorly, to make room for implant placement. This technique is more invasive and complex than lateralization, and while it is effective, it comes with higher risks, including a greater likelihood of permanent neurosensory disturbances. Studies have shown that transposition results in slower recovery rates than lateralization, with up to 25–40% of patients experiencing transient disturbances and some requiring up to 12 months for meaningful sensory improvement [3,31]. The incidence of permanent disturbances is also higher, with up to 8% of cases experiencing lasting sensory changes. Despite these drawbacks, transposition can be a viable option for patients with significant bone resorption or complex anatomy where lateralization may not be possible.

### 4.9. Comparison of Techniques

The choice of technique for rehabilitating the atrophic mandible depends on several factors, including the severity of bone atrophy, the patient’s overall health, the proximity of the inferior alveolar nerve, and the surgeon’s expertise.

### 4.10. Lateralization vs. Transposition

Lateralization was highlighted as superior in terms of sensory recovery and transient neurosensory disturbances by 12 studies. Recovery rates with lateralization exceeded 90% within six months, with seven studies reporting recovery rates of up to 95%. Recovery was notably faster, with significant improvement reported as early as 3 months [3,14,15,22].

Transposition, in contrast, resulted in slower recovery rates, averaging 75% within six months, and a higher incidence of permanent disturbances, reported in up to 8% of cases [3,14,31].

### 4.11. Technological Advances in Mandibular Rehabilitation

The introduction of piezosurgery and advancements in CBCT imaging have significantly improved the precision of both lateralization and transposition procedures. Piezosurgery, in particular, has been shown to offer superior outcomes in terms of sensory recovery due to its ability to minimize thermal damage and reduce surgical trauma [38]. Additionally, the use of CBCT imaging allows for better preoperative planning by providing detailed 3D views of the mandible and surrounding structures, ensuring more accurate nerve localization and bone measurement.

### 4.12. Neurosensory Disturbances

Neurosensory disturbances were a frequently reported outcome in procedures involving the IAN. Across the reviewed studies, transient disturbances were observed in 15–40% of cases, while permanent disturbances occurred in up to 5% of cases in procedures like lateralization and up to 8% in transposition [3,14,15,22,31].

The types of disturbances reported are as follows:

1. Paresthesia: The most common disturbance, characterized by tingling, numbness, or a “pins and needles” sensation [3,31].
2. Dysesthesia: Involving abnormal and unpleasant sensations [31,38].
3. Anesthesia: Loss of sensation, often temporary postoperatively [3,14].
4. Hyperesthesia: Over-sensitivity, though less common [3,15].

These disturbances often arise due to direct nerve trauma, thermal damage from rotary tools, ischemia caused by prolonged manipulation, or neural stretching and compression during nerve repositioning [15,31,38]. It was noted that subjective self-reports of neurosensory changes often lacked consistency, and objective tests like two-point discrimination were not uniformly applied across studies, further complicating comparisons [33,35].

### 4.13. Adjunctive Measures

Studies report varying incidences of NSDs, often transient, with recovery typically occurring within six to twelve months postoperatively. Factors influencing these outcomes include the surgical technique, the extent of nerve mobilization, and the use of adjunctive measures such as platelet-rich fibrin (PRF) conduits or piezoelectric devices [1,5]. For instance, the use of PRF has demonstrated promising results in accelerating nerve healing and reducing the duration of sensory deficits [5]. Similarly, with its precision and minimal thermal damage, piezosurgery has minimized intraoperative trauma and postoperative complications compared to conventional rotary instruments [1,2].

Piezosurgery demonstrated better neurosensory recovery, as noted in 14 studies, with recovery rates of 85–100% within three to six months [3,14,15,31]. Rotary tools were linked to slower recovery rates, with 25–40% of cases experiencing transient disturbances and delayed healing [3,32].

### 4.14. Feasibility of the Techniques

Lateralization using piezosurgery was identified as a highly feasible technique, with implant survival rates exceeding 97% and sensory recovery in 85–100% of cases within six months [3,14,15]. However, complications such as mandibular fractures were reported in some studies, highlighting the importance of careful patient selection and planning [3,14].

### 4.15. Clinical Implications

These findings emphasize the clinical importance of choosing between lateralization and transposition based on neurosensory risk. The main contribution of this review is to highlight that while both techniques are effective, transposition tends to carry a higher burden of transient sensory complications and slower recovery.

Based on current research, both transpositioning and inferior alveolar nerve lateralization are effective techniques for dental implant placement, showing comparable outcomes in terms of implant stability, marginal bone loss, and neurosensory disturbances. While the transpositioning approach has demonstrated slightly higher success rates and fewer neurosensory disturbances, these differences are not statistically significant. Immediate loading after relocation of the inferior alveolar nerve was successful in a recent case series [57].

### 4.16. Alternative Strategies and Future Perspectives

Alternatives such as shorter implants and tilted implants have been explored to avoid nerve repositioning procedures. Short implants (≤6 mm) have shown similar 5-year survival rates compared to longer implants (≥10 mm) in non-augmented bone and full-arch prostheses, indicating their viability in cases with limited bone height [35,56].

Tilted implants have also been studied, particularly in the rehabilitation of edentulous patients. Research indicates that tilted distal implants exhibit clinical effectiveness comparable to axial implants, aiding in posterior positioning without necessitating extensive bone augmentation [36,37].

Even though bone augmentation and the use of bone grafting have been reported to be effective surgical treatments, the risk of complications is quite high [58,59].

Moreover, the concept of “perigraftitis”, a type of biomedical device-associated infection (BAI), is increasingly recognized and investigated in the literature. Bone grafting has been identified as a significant risk factor for implant failure in the context of early infection. Implants placed in conjunction with bone grafts show lower survival rates following infection, as graft materials may act as foreign bodies, exacerbating the inflammatory response [60,61]. Other well-documented risk factors for implant failure include smoking (the most strongly associated), the use of bone grafts (which increases the likelihood and severity of infection), delayed diagnosis due to inadequate maintenance and oral hygiene (both at home and professionally), placement in post-extraction sockets, and immediate loading protocols [60,61].

An important clinical consideration of these relocation techniques may be that they are graft-less treatments, which are more effective in terms of long-term survival rates and have fewer complications and lower morbidity; this includes other surgical techniques using native bones in view of the medical principle of “less is more” [62,63].

The use of short implants, narrow implants, lamina implants, and subperiosteal implants could be an alternative in some cases [46,47,48,62,63,64].

### 4.17. Limitations

While this study provides valuable insights, certain limitations should be noted. The evaluation of neurosensory disturbances relied primarily on subjective self-reports and clinical observations, as objective tests such as evoked potentials were not utilized, which might have offered additional precision in assessing nerve function. The included studies were published between 2009 and 2024, excluding previous articles even if the technique of lateralization and transposition had already been used. The lack of modern instruments, such as piezosurgery or the precision of pre-surgical 3-D evaluation, was evaluated as a valid reason to exclude articles considering the year of publication. Variability in study design, sample size, follow-up duration, and patient characteristics introduced some heterogeneity; however, these differences reflect the diverse clinical contexts in which these techniques are applied. Although high-quality randomized controlled trials were limited, the included studies provide a robust foundation for understanding current practices. Future research with standardized protocols, larger sample sizes, and longer follow-up durations will further strengthen these findings and expand the evidence base for atrophic mandibular rehabilitation techniques. The statistical power of the meta-analysis is limited, as most pooled analyses were based on only two studies. This introduces wide confidence intervals and limits generalizability. These analyses should be considered as exploratory, and future studies with larger sample sizes are required to validate the trends observed here.

## 5. Conclusions

Atrophic mandibular rehabilitation remains a challenging but highly feasible procedure with the development of advanced techniques like nerve lateralization, transposition, and vertical bone augmentation. Lateralization is generally preferred due to its higher success rates and lower complication rates, while transposition remains an option for more complex cases. Vertical bone augmentation remains effective for restoring height in cases of severe bone loss but is associated with higher surgical complexity.

The continued development of surgical tools and imaging techniques, such as piezosurgery and CBCT, is likely to improve the precision and outcomes of these procedures. Future research is also warranted to evaluate the long-term outcomes and biomechanical stability of short implants and tilted implants as alternative strategies for managing atrophic mandibular cases. These approaches hold promise in reducing the need for extensive surgical interventions, particularly in patients with severe bone deficiencies or systemic conditions limiting their candidacy for traditional techniques.

Ultimately, patient selection and surgeon expertise remain critical factors in achieving optimal outcomes in atrophic mandibular rehabilitation.

## Figures and Tables

**Table 1:** Characteristics of included studies.

Author and Year	Study Design	Sample Size	Statistical Analysis Used	Radiographic Method	Method of Nerve Repositioning/Surgical Technique	Number of Patients with Altered Sensation	Method of Evaluation of Altered Sensation	Nature of Altered Sensation	Recovery Rate and Intervals	Implant Survival Rate
**Abdo et al., 2021** [14]	RCT	26 patients	Chi-square, *t*-test	CBCT	IAN lateralization vs. nerve bypass using computer-guided stent and conventional rotary method	26 patients	Subjective (questionnaire, light touch [LT], brush direction detection [BDD]), objective (TSEP)	Temporary sensory disturbance	Full recovery by 24 weeks	Not explicitly mentioned
**Campos et al., 2019** [15]	RCT	34 patients, 82 implants	Student’s *t*-test, ANOVA	CBCT	IAN lateralization with/without bone graft	34 patients	Questionnaire, periodic follow-up	Temporary hypoesthesia, paresthesia	Mean recovery: 118.6 days (control), 123.5 days (bone graft)	97.56%
**Metawie et al., 2022** [16]	RCT	20 patients (10 per group)	ANOVA, *t*-test	CBCT	IAN lateralization with sticky bone vs. bone block repositioning	20 patients	Subjective and objective testing, Modified Nerve Block Recovery (MNBR) scale	Temporary disturbance	100% recovery at 6 months	100%
**Garoushi et al., 2021** [17]	Prospective Randomized Clinical Trial	18 patients (30 ridges)	Chi-square, GEE model, *t*-test	CBCT	IAN lateralization with/without collagen membrane and bone graft	All patients initially	Medical Research Council (MRC) scale, sensory tests	Temporary disturbance	100% recovery at 6 months	100%
**chehata et al., 2021** [2]	Comparative Clinical Study	24 patients (12 per group)	*t*-test, ANOVA	CBCT	IAN lateralization with Piezosurgery vs. rotary instruments	24 patients	Transcutaneous electrical stimulation potential (TSEP), Visual Analog Scale (VAS)	Temporary disturbance	Full recovery by 24 weeks	95%
**Sethi et al., 2017** [18]	Retrospective Analysis	78 patients, 308 implants	Kaplan–Meier survival curve	CBCT	IAN lateralization/transposition	All initially; 5 patients with residual altered sensation	Wisp test, sharp test, discriminatory distance test	Residual altered sensation in 5 patients	Recovery varied between 24 h and 6 months	97.8%
**Bayram et al., 2023** [19]	Prospective Cohort Study	20 patients, 50 implants	Shapiro–Wilk, ANOVA, Bonferroni post hoc	CBCT	IAN lateralization	20 patients initially	Westermark’s subjective method, Von Frey hair test	Temporary paresthesia	Median duration 120 days, no permanent issues at 12 months	100%
**De Vicente et al., 2016** [1]	Prospective Clinical Study	13 patients, 27 implants	Mann–Whitney, Fisher’s exact test, Spearman’s correlation	CT scans	IAN lateralization with buccal cortical bone repositioning	All patients initially	Light touch (LT), pain threshold, Two-point discrimination (TPD) tests	Temporary hypoesthesia	11 fully recovered at 3 months, 1 patient had residual sensation at 12 months	100%
**Dursun et al., 2016** [20]	Prospective Comparative Study	15 patients (25 implants in IANL group)	ANOVA, Pearson Chi-square test	CBCT, Panoramic X-rays	IAN lateralization with Piezosurgery vs. short implant conventional methods	2 patients	Two-point discrimination, pin-prick tests	Temporary paresthesia	Resolved in 1 week	100%
**Fernández et al., 2013** [21]	Prospective Cohort Study	15 patients (19 procedures)	Descriptive statistics	Panoramic X-rays	IAN lateralization with Piezosurgery	All patients initially	Two-point discrimination test	Temporary hypoesthesia	93.33% recovered at 8 weeks	97.36%
**Hashemi et al., 2010** [22]	Prospective Cohort Study	87 patients (110 sites)	Descriptive statistics	Panoramic X-rays, CT scans	IAN lateralization using hand instruments	All patients initially	Questionnaire (subjective reporting of sensory disturbances)	Temporary hypoesthesia, tickling in 3% at 6 months	97% normal neurosensory function by 1 year	100%
**Martínez-Rodríguez et al., 2016** [23]	Prospective Cohort Study	27 patients, 74 implants	Descriptive statistics	Panoramic radiographs, CT scans	IAN lateralization with Piezosurgery	27 patients initially	Two-point discrimination test	Temporary hypoesthesia	74.1% recovery at 3 months; 96.3% recovery at 18 months	98.6% preloading, 100% post-loading
**Rathod et al., 2018** [24]	Prospective Clinical Study	10 patients, 20 implants	Descriptive statistics	CBCT, Panoramic radiographs	IAN lateralization	All patients initially	Semmes–Weinstein monofilaments (SWMs)	Temporary hypoesthesia	Minimum recovery time: 2 months; maximum: 4 months	Not reported
**Saad Al-Almaie et al., 2020** [25]	Prospective Study	8 patients, 20 implants	Life-table analysis	Panoramic radiographs	IAN transposition	6 patients initially	Light touch test, pain test, two-point discrimination test	Temporary neurosensory disturbances in 20% of cases	Complete recovery in 3 patients within 1 month	100%
**Castellano-Navarro et al., 2019** [26]	Retrospective Case Series	123 patients, 337 implants	Descriptive statistics	Panoramic X-rays	IAN lateralization and transposition	All patients initially	Light touch test, sensitivity mapping	Temporary hypoesthesia in all patients	81% recovery within 6 months, 100% by 1 year	Not reported
**Gasparini et al., 2014** [3]	Retrospective Cohort Study	35 patients, 49 IANTs	Fisher’s exact test	CT dentascan	IAN transposition	6 patients with complications	Two-point discrimination test, painful stimulus, thermal sensitivity	Transient hypoesthesia (14.3%) and anesthesia (2.8%)	All symptoms resolved by 6 months	Not reported
**Deryabin et al., 2021** [6]	Retrospective Multicenter Study	15 patients, 48 implants	Descriptive statistics	CBCT	IAN lateralization and transposition	All patients initially	Subjective assessment using a modified questionnaire	Transient numbness in all patients; weak hypoesthesia in 2 patients by 3 years	Transient numbness resolved in most cases by 3 months; weak hypoesthesia persisted in 2 patients at 3 and 5 years	95.8%
**Khojasteh et al., 2016** [5]	Retrospective Cohort Study	14 patients, 51 implants	Descriptive statistics	CBCT, Panoramic X-rays	Modified IAN lateralization with PRF conduit	All patients initially	Static light touch (SLT) and two-point discrimination (TPD) tests	Numbness, tingling; transient hypoesthesia	Normal sensation at 6 months in 42.9% (modified) and 28.6% (conventional); full recovery in most by 12 months	Not explicitly reported
**Lorean et al., 2013** [4]	Multicenter Retrospective Study	57 patients, 232 implants	Descriptive statistics	CT scans	IAN transposition/reposition	4 patients (5%)	Von Frey test, two-point discrimination, pin-prick tests	Prolonged transient neural disturbance (1–6 months)	No permanent neural damage; full recovery by 6 months in most cases	99.57%
**Nishimaki et al., 2016** [27]	Retrospective Assessment	7 patients, 22 implants	Descriptive statistics	CBCT, Panoramic X-rays	IAN transposition	All patients initially	Modified SW perception test, highest grading	Transient numbness, moderate hypoesthesia, severe hypoesthesia	Full recovery on 2 sides; weak hypoesthesia in 2 sides; moderate in 2 sides; severe in 1 side	100%

**Table 2:** Cochrane risk of bias tool for randomized trials (Rob 2).

Study	Bias from Randomization	Bias from Interventions	Bias from Missing Data	Bias from Outcome Measurement	Bias from Reported Results	Overall Risk of Bias
Abdo et al. [14]	Low Risk	Low Risk	Low Risk	Low Risk	Low Risk	Low Risk
Campos et al. [15]	Low Risk	Low Risk	Low Risk	Low Risk	Low Risk	Low Risk
Metawie et al. [16]	Low Risk	Low Risk	Low Risk	Low Risk	Low Risk	Low Risk
Garoushi et al. [17]	Low Risk	Low Risk	Low Risk	Low Risk	Low Risk	Low Risk
chehata et al. [2]	Low Risk	Low Risk	Low Risk	Low Risk	Low Risk	Low Risk

**Table 3:** Newcastle–Ottawa scale for retrospective studies (NOS) in wich the Selection, Comparability and Outcome were evaluated as bad with “★”, medium “★★”, medium well structured “★★★” and optimum “★★★★”.

Study	Selection (4)	Comparability (2)	Outcome (3)	Total (9)
Lorean et al. (2013) [4]	★★★★	★★	★★★	9/9 (Low Risk)
Sethi et al. (2017) [18]	★★★★	★★	★★★	9/9 (Low Risk)
Castellano et al. (2019) [26]	★★★★	★	★★★	8/9 (Low Risk)
Gasparini et al. (2014) [3]	★★★★	★★	★★	8/9 (Low Risk)
Nishimaki et al. (2016) [27]	★★★★	★★	★★	8/9 (Low Risk)
George Deryabin et al. (2021) [6]	★★★	★	★★	6/9 (Moderate Risk)
Khojasteh et al. (2016) [5]	★★★	★	★★	6/9 (Moderate Risk)

**Table 4:** ROBINS-I risk of bias tool for non-randomized studies.

Study	Confounding Bias	Selection Bias	Intervention Classification Bias	Deviation Bias	Missing Data Bias	Measurement Bias	Reporting Bias	Overall Risk of Bias
Bayram et al. [19]	Low	Low	Low	Low	Low	Low	Low	Low
De Vicente et al. [1]	Moderate	Low	Low	Moderate	Low	Low	Low	Moderate
Erhan Dursun et al. [20]	Moderate	Moderate	Low	Moderate	Moderate	Moderate	Moderate	Moderate
Fernandez Diaz et al. [21]	Low	Low	Low	Low	Low	Low	Low	Low
Hashemi et al. [22]	Moderate	Moderate	Low	Moderate	Moderate	Moderate	Moderate	Moderate
Martínez-Rodríguez et al. [23]	Low	Low	Low	Low	Low	Low	Low	Low
Rathod et al. [24]	Moderate	Moderate	Low	Moderate	Moderate	Moderate	Moderate	Moderate
Saad Al-Almaie et al. [25]	Moderate	Moderate	Low	Moderate	Moderate	Moderate	Moderate	Moderate

**Table 5:** List of Full-Text Articles Excluded with Reasons.

**Article citation**	Reason for Exclusion
**Meyer et al.** [28]	Letters to editor
**Robinson et al.** [29]	No comparison between lateralization and transposition
**Hirsch et al.** [30]	Year of publications
**Palacio García-Ochoa et al.** [31]	
**Felice et al.** [32]	Only evaluated short implants and bone augmentation, no nerve relocation
**Allavéna et al.** [33]	Narrative review, not original data
**Valenzuela-Fuenzalida et al.** [34]	Narrative review
**Abayev et al.** [35]	
**Ravid et al.** [36]	Systematic review and no nerve technique evaluated
**Mehta et al.** [37]	Systematic review and no nerve technique evaluated
**Aiuto et al.** [38]	Study on alternative procedures
**Vetromilla et al.** [39]	No comparison between lateralization and transposition
**Yoshimoto et al.** [40]	Technology report, no clinical outcomes
**Turhani et al.** [41]	Narrative review with case report
**Van Vo et al.** [42]	Case report
**Louis et al.** [43]	No implant placement
**Vatteroni et al.** [44]	Review article of alternative procedures
**Libertucci et al.** [45]	No nerve relocation data
**Toti et al.** [46]	Clinical article of alternative procedures
**Valente et al.** [47]	Clinical article of alternative procedures
**Vercellotti et al.** [48]	Clinical article of alternative procedures
**Vinci et al.** [49]	Technical review without clinical data related to relocation of the nerve
**Tereshchuk et al.** [50]	No implant placement
**Hassani et al.** [51]	Technical method description
**Romanos et al.** [52]	Technical method description
**Suzuki et al.** [53]	Case report
**Morrison et al.** [54]	It has not been published between 2009 and 2024
**Zandi et al.** [55]	Animal study
